# Promoter-Specific Hypomethylation Correlates with IL-1β Overexpression in Tuberous Sclerosis Complex (TSC)

**DOI:** 10.1007/s12031-016-0750-7

**Published:** 2016-04-28

**Authors:** A. Fuso, A. M. Iyer, J. van Scheppingen, M. Maccarrone, T. Scholl, J. A. Hainfellner, M. Feucht, F. E. Jansen, W. G. Spliet, P. Krsek, J. Zamecnik, A. Mühlebner, E. Aronica

**Affiliations:** 1 European Center for Brain Research (CERC)/IRCCS Santa Lucia Foundation, Via del Fosso di Fiorano 64-65, 00143 Rome, Italy; 2 Department of (Neuro)Pathology, Academic Medical Center, University of Amsterdam, Meibergdreef 9, 1105 AZ Amsterdam, The Netherlands; 3 Swammerdam Institute for Life Sciences, Center for Neuroscience, University of Amsterdam, Amsterdam, The Netherlands; 4 Department of Medicine, Campus Bio-Medico University of Rome, Rome, Italy; 5 Department of Pediatrics, Medical University Vienna, Vienna, Austria; 6 Institute of Neurology, Medical University Vienna, Vienna, Austria; 7 Department of Pediatric Neurology, Brain Center Rudolf Magnus, University Medical Center Utrecht, Utrecht, The Netherlands; 8 Department of Pathology, University Medical Center Utrecht, Utrecht, The Netherlands; 9 Department of Pediatric Neurology, 2nd Faculty of Medicine, Charles University, Motol University Hospital, Prague, Czech Republic; 10 Department of Pathology, 2nd Faculty of Medicine, Charles University, Motol University Hospital, Prague, Czech Republic; 11 Stichting Epilepsie Instellingen Nederland (SEIN), Heemstede, The Netherlands

**Keywords:** Tuberous sclerosis complex, Cortical tuber, Inflammation, Interleukin-1β, Epigenetic regulation

## Abstract

In tuberous sclerosis complex (TSC), overexpression of numerous genes associated with inflammation has been observed. Among different proinflammatory cytokines, interleukin-1β (IL-1β) has been shown to be significantly involved in epileptogenesis and maintenance of seizures. Recent evidence indicates that IL-1β gene expression can be regulated by DNA methylation of its promoter. In the present study, we hypothesized that hypomethylation in the promoter region of the IL-1β gene may underlie its overexpression observed in TSC brain tissue. Bisulfite sequencing was used to study the methylation status of the promoter region of the IL-1β gene in TSC and control samples. We identified hypomethylation in the promoter region of the IL-1β gene in TSC samples. IL-1β is overexpressed in tubers, and gene expression is correlated with promoter hypomethylation at CpG and non-CpG sites. Our results provide the first evidence of epigenetic modulation of the IL-1β signaling in TSC. Thus, strategies that target epigenetic alterations could offer new therapeutic avenues to control the persistent activation of interleukin-1β-mediated inflammatory signaling in TSC brain.

## Introduction

Tuberous sclerosis complex (TSC) is a multisystem genetic disorder that results from a mutation in the TSC1 or TSC2 genes leading to constitutive activation of mammalian target of rapamycin complex 1 (mTORC1) and is therefore highly associated with intractable epilepsy (Curatolo et al. 2015). Cortical tubers are believed to represent the neuropathological substrate in TSC patients. However, a growing body of evidence supports the existence of a more extensive epileptogenic network in TSC patients (Marcotte et al. 2012; Okanishi et al. 2014). Cortical tubers, but to a certain extent also the perituberal cortex, are characterized by a complex activation of pro-inflammatory signaling pathways, including, in particular, the IL-1β signaling pathway (Boer et al. 2010; Boer et al. 2008). Expression of both IL-1β and its receptor was observed in astrocytes, cells of the microglia/macrophage lineage, dysmorphic neurons, and giant cells displaying mTOR activation (Boer et al. 2008). Evaluation of fetal TSC cases demonstrated an activation of the IL-1 receptor (R)/Toll-like receptor (TLR) pathway also in developing TSC brain lesions (Prabowo et al. 2013), suggesting that the induction of this pathway could be intrinsic to the developmental lesion and linked to the deregulation of the mTOR pathway.

Recent evidence suggests that pro-inflammatory cytokines, including IL-1β, can be epigenetically regulated through DNA methylation at their promoters (Aoi et al. 2011; Hashimoto et al. 2013; Kirchner et al. 2014; Tekpli et al. 2013). In particular, a recent study shows that, in mice and humans, hypomethylation of IL-1β at specific CpG sites is associated with elevated IL-1β transcription. Hence, this epigenetic mechanism may contribute to cognitive deficits in aging and neurodegenerative diseases (Cho et al. 2015). The influence of epigenetic regulation of IL-1β in TSC brain remains as yet unclear. We hypothesized therefore that hypomethylation in the promoter region of the IL-1β gene may underlie its overexpression observed in tubers.

## Patients and Methods

### Patient Cohort

The cases included in this study were obtained from the archives of the departments of neuropathology of the Academic Medical Center (AMC, University of Amsterdam), University Medical Center in Utrecht (UMCU), Motol University Hospital (Prague, Czech Republic), and Medical University Vienna (Vienna, Austria). We evaluated five tubers (three surgical and two autopsy specimens; mean age, 15.8 years; male/female, 2/3; localization, frontal/temporal/parietal, 3/1/1; TSC1/TSC2, 1/4). Four cases (two autopsy/two surgical; male/female, 1/3; mean age: 18.4 years; TSC1/TSC2:1/3) contained sufficient amount of perituberal tissue, defined by the absence of dysmorphic neurons and giant cells. The age- and localization-matched control group consisted of six autopsy cases (male/female, 2/4; mean age, 13.5 years; frontal/temporal/parietal, 4/1/1). None of these patients had a history of seizures or other neurological diseases.

Tissues were obtained and used in accordance with the Declaration of Helsinki and the AMC Research Code provided by the Medical Ethics Committee. The local ethical committees of all participating centers gave permission to undertake the study.

### Tissue Preparation

Brain tissues from control autopsy patients and TSC patients were snap frozen in liquid nitrogen and stored at −80 °C until further use. Formalin-fixed paraffin-embedded tissues were sectioned at 6 μm, mounted on pre-coated glass slides (Star Frost, Waldemar Knittel GmbH, Braunschweig, Germany). Immunohistochemistry for IL-1β (polyclonal goat antibody; sc-1250, Santa Cruz Bio., CA, USA; 1:70) was carried out on paraffin-embedded tissue as previously described (Boer et al. 2008; Ravizza et al. 2006). Sections were deparaffinated in xylene, rinsed in ethanol (100, 95, and 70 %), and incubated for 20 min in 0.3 % hydrogen peroxide diluted in methanol. Antigen retrieval was performed using a pressure cooker in 0.1 M citrate buffer pH 6.0 at 120 °C for 10 min. Slides were washed with phosphate-buffered saline (PBS; 0.1 M, pH 7.4) and incubated overnight with the primary antibody in PBS at 4 °C. After washing in PBS, sections were incubated with rabbit anti-goat IgG (hum absorb, SBA 6164–01, 1:2000, 15 min), subsequently with polymer goat anti-rabbit IgG/HRP (BrightVision, DPVM-15HRP, undiluted, 30 min) and stained using 3,3′-diaminobenzidine tetrahydrochloride as chromogen. Sections were counterstained with hematoxylin, dehydrated in alcohol and xylene, and coverslipped. The specificity of the antibody was tested by pre-incubating the antibody with a 100-fold excess of the antigenic peptides (Santa Cruz Bio. and R&D Systems). Sections incubated without the primary Ab or with the primary Ab and an excess of the antigenic peptide were essentially blank (Ravizza et al. 2006). Paraffin-embedded human specimens of gliomas (Giometto et al. 1996; Sasaki et al. 1998), multiple sclerosis (Huitinga et al. 2000), and viral encephalitis (herpes simplex encephalitis and rabies encephalitis) were used as positive controls for immunocytochemical staining. For double-labeling studies, sections were incubated with the primary antibodies (anti-IL-1β and anti-GFAP; monoclonal mouse, Sigma, St. Louis, Mo, USA; 1:4000) and were incubated for 2 h at RT with Alexa Fluor® 568-conjugated anti-goat IgG and Alexa Fluor® 488 anti-mouse, IgG (1:200, Molecular Probes, The Netherlands). Sections were then analyzed by means of a laser scanning confocal microscope (Leica TCS Sp2, Wetzlar, Germany).

### DNA Methylation Analysis by Bisulfite Modification and Genomic Sequencing

**Fig. 1:**
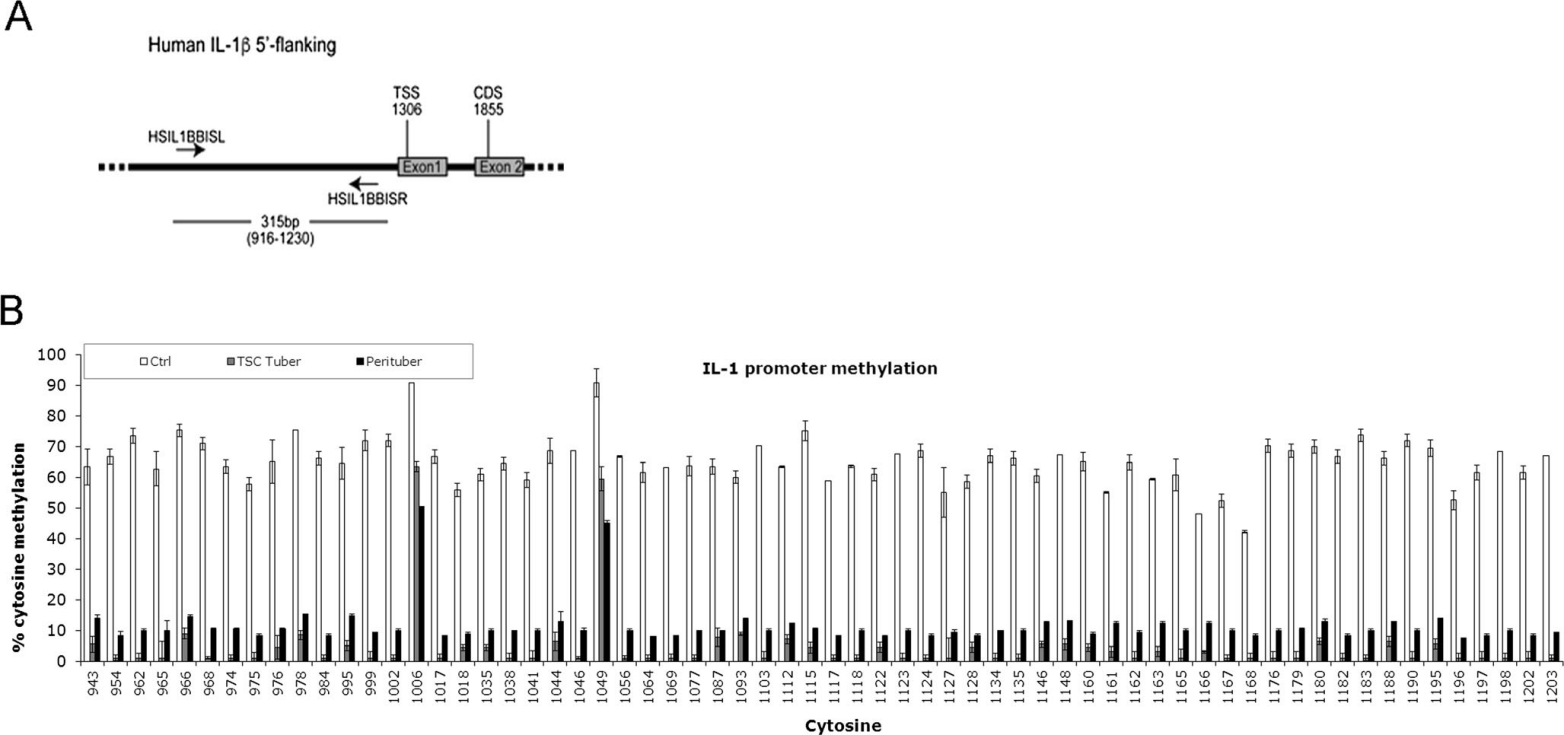
Methylation pattern of IL-1β promoter in controls and TSC brain tissue. **a** Schematic representation of the 5′-flanking region on the *IL-1β* gene, reporting the position of primers used in the bisulfite analysis and the position of the transcription start site (*TSS*) and the start site of the coding sequence (*CDS*). **b** CpG and non-CpG site-specific methylation pattern expressed as percent methylation for each cytosine in the investigated region of the human *IL-1β* promoter. Cytosine position in the reference sequence is indicated below the *x*-axis. *White columns* represent control samples, *gray columns* represent tuber samples, and *black columns* represent perituberal tissue samples

DNA was extracted from frozen tissue material of controls and TSC patients using the QIAamp DNA Mini Kit (Qiagen, Venlo, The Netherlands). DNA methylation analysis was carried out as previously described (Fuso et al. 2015). Briefly, bisulfite analysis of *IL-1β* promoter methylation was performed using the EpiTect Bisulfite kit; PCR products obtained after bisulfite treatment were cloned using the PCR Plus Cloning Kit (both from Qiagen). At least ten clones were analyzed per experimental condition using M13 primers for sequencing performed by Primm (Milan, Italy). The primers used for the amplification of the bisulfite-modified DNA were designed on the GenBank AY137079.1 sequence, considering the 5′-flanking region (Fig. 1a) of the messenger RNA (mRNA) (TSS at the base n. 1306). These primers (HSIL1BBISL, 5′-AAAGAAGTGAATGAAGAAAAGTATGTG-3′; HSIL1BBISR, 5′-AATACCTRATTTCACAATCAARTTAAA-3′) amplified a 315-bp sequence (916–1230), containing 64 cytosines including two CpG moieties (1006 and 1049). These primers are “methylation insensitive primers” (MIPs) that can detect methylation in both CpG and in non-CpG moieties (Fuso et al. 2015) and allow assessing methylation status of plus (5′-3′) DNA strand.

As negative controls of bisulfite modifications, we used unmethylated purified PCR product of *IL-1β* promoter obtained from genomic DNA by the same primers used for the amplification of the bisulfite-treated DNA. The same purified PCR product was methylated in vitro with SssI methylase (New England Biolabs, EuroClone, Milan, Italy) and was used as positive controls.

Modified cytosine residues were recognized through comparison with the original DNA sequence, and methylation status of any single cytosine in each sequenced clone was reported as 1/0 value in an Excel spreadsheet (methylated, 1; unmethylated, 0). For each experimental sample, we obtained the methylation percentage of each single cytosine by calculating the number of methylated cytosines divided by the number of clones sequenced per 100 ([no. methylC/no. sequenced clones] × 100; (Fuso et al. 2015)).

### RNA Isolation and Real-Time Quantitative PCR Analysis

For RNA isolation, frozen material was homogenized in Qiazol Lysis Reagent (Qiagen Benelux, Venlo, the Netherlands). Total RNA was isolated using the miRNeasy Mini kit (Qiagen Benelux, Venlo, the Netherlands). The concentration and purity of RNA were determined using a Nanodrop 2000 spectrophotometer (Thermo Scientific, Wilmington, DE, USA). To evaluate the expression of IL-1β mRNA in control and TSC tissues, 5 μg of total RNA was reverse-transcribed into complementary (cDNA) using oligo dT primers. Specific primers were designed using the Universal ProbeLibrary Assay Design Center of Roche (https://www.roche-applied-science.com) on the basis of the reported mRNA sequences. The following primers were used: IL-1β (forward: gcatccagctacgaatctcc reverse: gaaccagcatcttcctcagc, product size 99 nt); elongation factor 1-alpha (EF1α; forward: atccacctttgggtcgcttt; reverse: ccgcaactgtctgtctcatatcac, product size 51 nt). Qualitative real-time PCR (qRT-PCR) and quantification was performed as previously described (Prabowo et al. 2015). Briefly, for 1 μl cDNA, a master mix was prepared containing 2X SensiFAST^™^ SYBR No-ROX (Bioline, Taunton, MA, USA) forward and reverse primers in a total volume of 5 μl. The samples were run in triplicate in a 384-well plate in the LightCycler® 480 Real-Time PCR System (Roche Applied Sciences) under the following conditions: a 2-min denaturing step at 95 °C followed by a total of 45 amplification cycles consisting of 5 s of denaturing at 95 °C, 10 s of annealing at 65 °C, and 15 s of extension at 72 °C. Fluorescent product was measured by a single acquisition mode at 72 °C after each cycle. Quantification of data was performed using the computer program LinRegPCR in which linear regression on the Log (fluorescence) per cycle number data is applied to determine the amplification efficiency per sample as described (Ramakers et al. 2003; Ruijter et al. 2009). The starting concentration of each specific product was divided by the starting concentration of reference gene (elongation factor 1-alpha (EF1)), and this ratio was compared between groups. The results were expressed as fold change with respect to control values.

### Statistical Analysis

Statistical analyses were performed using the GraphPad Prism® software (GraphPad software Inc., La Jolla, CA, USA). The non-parametric Kruskal-Wallis test followed by pairwise comparison was used to analyze DNA methylation differences and the expression of IL-1β between multiple groups. The correlation between DNA methylation and IL-1β gene expression was analyzed by Spearman’s rank correlation analysis.

## Results

All the five tubers displayed similar histopathological features with astrogliosis, loss of lamination, giant cells, and dysmorphic neurons.

Significant overall hypomethylation of the *IL-1β* promoter was observed between the groups [Kruskal-Wallis H[2] = 10.485, *p* < 0.01]. However, pairwise comparison revealed significant differences in methylation read counts in tubers and control tissue (*p* < 0.01) while a trend towards hypomethylation was seen in perituberal tissue compared to controls. The hypomethylation was particularly evident for non-CpG moieties (*p* < 0.001, tubers and perituberal tissues vs. control tissues) although the two CpG moieties (cytosines 1006 and 1049, *p* < 0.01) also showed significant hypomethylation compared to control tissues (Fig. 1b).

**Fig. 2:**
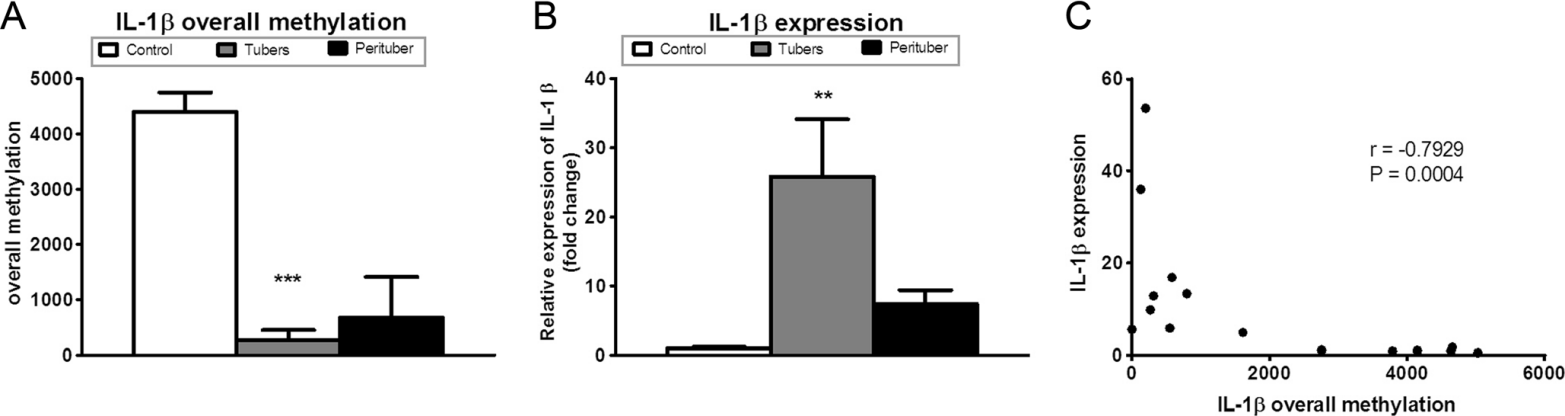
IL-1β promoter hypomethylation correlates with gene expression. **a** Overall sites of methylation (CpG and non-CpG) in the investigated region of the human *IL-1β* promoter. **b** Quantitative real-time PCR of IL-1β mRNA expression in TSC and control samples, expressed as fold change with respect to controls; *white columns* represent control samples, *gray columns* represent tuber samples, and *black columns* represent perituberal tissue samples. **c** Correlation between IL-1β DNA methylation (*x*-axis) and gene expression (*y*-axis). *r* = Spearman’s rank correlation coefficient, **p < 0.01, ***p < 0.001

Increased expression of IL-1β mRNA was observed in tubers (*p* < 0.01), whereas a trend to increased expression was observed in the perituberal cortex as compared to controls (Fig. 2b) by qRT-PCR. A significant inverse correlation was observed between the extent of DNA methylation and gene expression (Fig. 2c; *r* = −0.7929, *p* < 0.001) by Spearman’s rank correlation analysis.

**Fig. 3:**
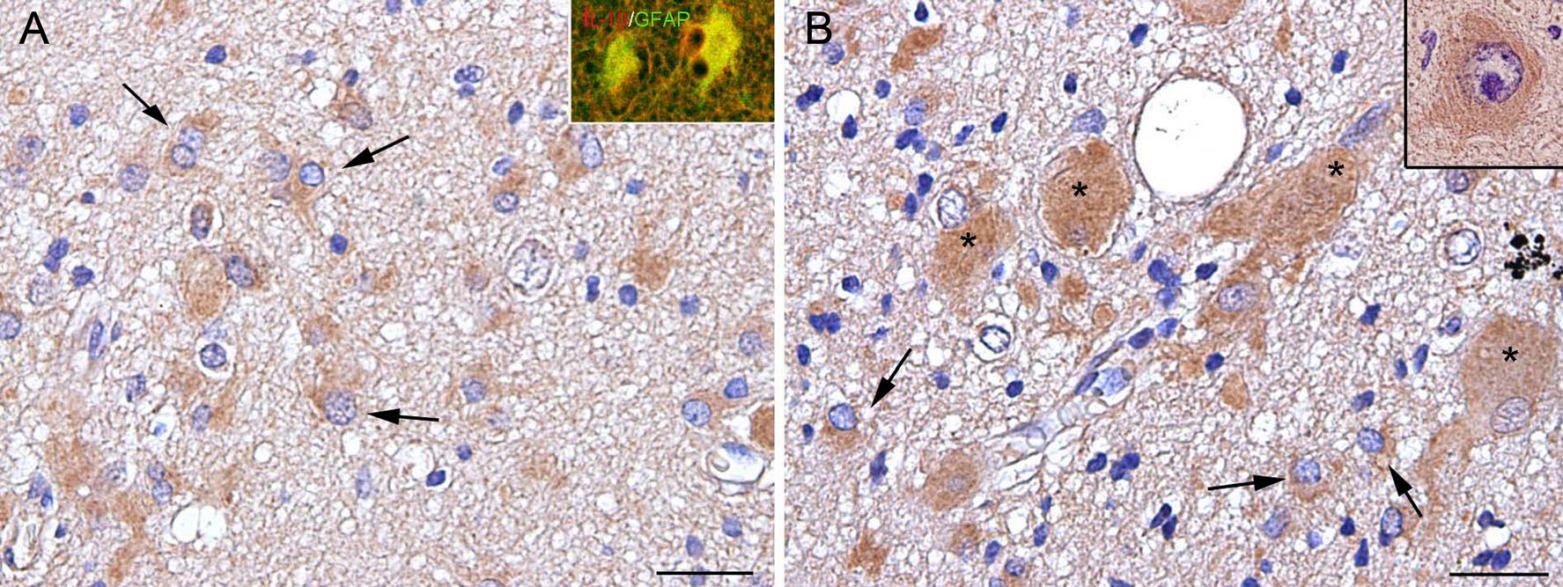
Expression of IL-1β immunoreactivity (IR) in cortical tubers. **a**, **b** IL-1β IR is observed in reactive astrocytes (*arrows*) and in giant cells (*asterisks*) in TSC brain (cortical tuber); *inset* in (**a**) is a merged image, showing expression of IL-1β in GFAP-positive cells; inset in (**b**) shows a positive dysmorphic neuron. *Scale bars* 40 μm

In agreement with previous reports (Boer et al. 2008; Ravizza et al. 2006), increased IL-1β expression was detected in TSC tubers as compared to control tissue; expression of IL-1β was observed in glial and neuronal cells, as well as in giant cells within the tuber (Fig. 3a, b).

## Discussion

Previous studies have identified altered gene expression in brain lesions typical for TSC, in particular, an enhanced expression of numerous genes associated with the immune and inflammatory response (for review, see Aronica and Crino (2014)). However, these studies have not provided insights into the underlying mechanisms of transcriptional dysregulation, such as aberrant epigenetic control through DNA hypo- and/or hypermethylation. In this study, we focused on IL-1β, a pro-inflammatory cytokine, strongly deregulated in TSC human brain and known to play a key pathogenic role in human epilepsy (Vezzani et al. 2013). Herein, we described for the first time evidence of significant *IL-1β* promoter hypomethylation in TSC brain specimens compared to controls. Noteworthy, the hypomethylation was particularly evident in non-CpG cytosine moieties. Our data clearly demonstrated that the increased IL-1β expression in tuber and perituberal TSC cortex correlated with the hypomethylation of the *IL-1β* gene promoter. The implication that also perituberal tissues maintain higher gene expression and lower promoter methylation than control tissues suggests that IL-1β methylation-mediated overexpression may represent an intrinsic feature of TSC brain, independent of tuber pathology visible on histology. A similar observation was reported in recent studies, using animal models, supporting the role of the TORC1 signaling in epilepsy development, even in the absence of major brain pathology (Abs et al. 2013), and demonstrating also specific molecular abnormality independent of tubers (Lozovaya et al. 2014). Moreover, a recent study shows over-activation of IL-1β signaling pathway in astrocytes before epilepsy onset in a mouse model of TSC, pointing to the role of mTOR-mediated inflammatory mechanisms in TSC (Zhang et al. 2015).

Further research is, however, needed to fully understand the mechanism of genomic hypomethylation (particularly promoter specific DNA methylation) in the pathogenesis of TSC and its contribution to the neurological manifestation of TSC patients. For example, the study of Cho et al. (2015) suggests that the glial levels of sirtuin 1 (SIRT1) could play a key role in initiating epigenetic alterations through hypomethylation of IL-1β, leading to its enhanced expression and cognitive decline. The role of SIRT1 as potential negative regulation of inflammation (Xie et al. 2013) has not yet been investigated in TSC. Moreover, several alternative mechanisms may contribute to IL-1β upregulation. There is increasing evidence of complex processes of transcriptional and post-transcriptional regulation of cytokines, including IL-1β (Auron and Webb 1994; Fenton 1992; Simi et al. 2007). IL-1β expression is regulated by components of the complement cascade, several cytokines (including and IL-1β itself; (Fenton 1992 Lucas, 2006 #33,912)), and damage-associated molecular pattern molecules, which include high-mobility group box 1 (HMGB1; (Pedrazzi et al. 2007)). Interestingly, cortical tubers are characterized by prominent activation of pro-inflammatory signaling pathways, including in particular the complement and Toll-like receptor pathways and HMGB1 signaling (Boer et al. 2010; Boer et al. 2008; Zurolo et al. 2011). Attention has been also recently focused on binding proteins and microRNAs, which may directly regulate the stability and/or translation of cytokine mRNAs (Palanisamy et al. 2012) and interact with IL-1β signaling pathway via complex positive and negative feedback loops. (He et al. 2014; O’Neill et al. 2011; Quinn and O’Neill 2011; van Scheppingen et al. 2016). Finally, since patients received several antiepileptic drugs, such as valproic acid, a possible effect of drug treatment on DNA methylation patterns (Ni et al. 2015), as well as on IL-1β expression (Gomez et al. 2014; Verrotti et al. 2001), has to be taken into consideration in the interpretation of the expression data in TSC brain specimens.

In summary, the presented data demonstrated the potential role of gene-specific DNA hypomethylation inducing aberrant transcriptional control that may lead to the increased expression of IL-1β under pathological conditions. Thus, strategies that target epigenetic alterations, combined with conventional therapies, could offer new therapeutic avenues to control the IL-1β-mediated signaling and to develop a more personalized treatment in TSC patients.
